# Composed query image retrieval based on triangle area triple loss function and combining CNN with transformer

**DOI:** 10.1038/s41598-022-25340-w

**Published:** 2022-12-02

**Authors:** Zhiwei Zhang, Liejun Wang, Shuli Cheng

**Affiliations:** College of Information Science and Engineering, Xinjiang University, Urumqi, 830046 China

**Keywords:** Engineering, Learning algorithms, Network models

## Abstract

The existing typical combined query image retrieval methods adopt Euclidean distance as sample distance measurement method, and the model trained by triple loss function blindly pursues absolute distance between samples, resulting in unsatisfactory image retrieval performance. Meanwhile, these methods singularly adopt Convolutional Neural Network (CNN) to extract reference image features. However, receptive field of convolution operation has the characteristics of locality, which is easy to cause the loss of edge feature information of reference images. In view of shortcomings of these methods, the following improvements are proposed in this paper: (1) We propose Triangle Area Triple Loss Function (TATLF), which adopts Triangle Area (TA) as measurement of sample distance. TA comprehensively considers the absolute distance and included angle between samples, so that the trained model has better retrieval performance; (2) We combine CNN with Transformer to simultaneously extract local and edge features of reference images, which can effectively reduce the loss of reference images information. Specifically, CNN is adopted to extract local feature information of reference images. Transformer is used to pay attention to the edge feature information of reference images. Extensive experiments on two public datasets, Fashion200k and MIT-States, confirm the excellent performance of our proposed method.

## Introduction

Researchers began study on image retrieval in the 1970s. Initially, text-based image retrieval (TBIR)^1^ was studied. TBIR requires humans to manually label images, which is inefficient and highly subjective. The new retrieval technology appeared in the 1990s: content-based image retrieval (CBIR)^2,3^. Since CBIR only extracts and analyzes the low-level features of the image, it is often very different from the “original intention” of query. In view of shortcomings of CBIR technology, semantic-based image retrieval (SBIR)^4^ technology has been proposed. SBIR considers not only the low-level features of the image, but also the high-level features of the image, such as spatial relationship, scene, and emotion.

Image retrieval is widely used in various fields, which can be divided into medical^5,6^ and non-medical fields^7,8^. In medical field, auxiliary diagnosis is carried out through image retrieval technology, which helps doctors to formulate treatment plans quickly and accurately. In non-medical field, image retrieval is used for information filtering, such as shopping and travel.

In recent years, single-modal image retrieval technology has been unable to meet retrieval requirements of users. How to quickly and accurately retrieve the image information required by users has become a research hot spot in the field of image retrieval. When the task of retrieving target images by reference images is performed, existing reference images cannot accurately express the inner thought of users, which eventually leads to the inability to retrieve “ideal” target images. Hence, Combined query image retrieval is gradually proposed to solve this problem.

**Figure 1:**
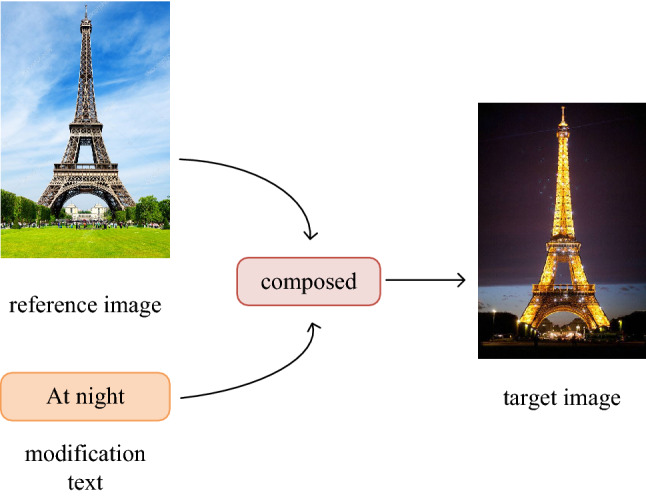
Example of image retrieval using reference image and text as query. (Created by ‘Microsoft Office Visio 2013’ https://www.microsoft.com/zh-cn/microsoft-365/previous-versions/microsoft-vision-2013).

The core of combined query image retrieval^9,10^ is describe a reference image through text, so as to achieve purpose of retrieving the target image. As shown in Fig. 1, there is a reference image of “Eiffel Tower at daytime”, but we want to find a target image of “Eiffel Tower at night”, so we need to describe the difference between the reference image and the target image through text. Specifically, we need to modify the reference image features by the text features. How to modify it? Where to modify it? These two research difficulties have been the focus of research in this field. In recent years, Vo et al. proposed the Text Image Residual Gating (TIRG) method^11^, which well solved problems of “how to modify” and “where to modify”. However, the sample distance measurement method in the TIRG adopts Euclidean distance, and model trained by triple loss function blindly pursues absolute distance between samples, resulting in poor image retrieval performance. Meanwhile, the TIRG only adopts CNN to extract reference image features. CNN extracts image features through convolution, but the inherent locality of receptive field of convolution operation can easily cause the loss of edge feature information of reference images. Therefore, it is difficult to match target features with combined features.

In view of the above shortcomings, the main contributions of this paper are as follows:

1. We propose Triangle Area Triple Loss Function (TATLF), which adopts TA as measurement of sample distance. TA considers not only the absolute distance between samples, but also the included angle between samples.
2. We combine CNN with Transformer to capture local and edge feature information of reference images, which can reduce the loss of information. Specifically, the local feature information of reference images is extracted by CNN. Meanwhile, the edge feature information of reference images is focused through Transformer.
3. Extensive experiments on two public datasets, Fashion200k and MIT-States, confirm the excellent performance of our proposed method. Taking R@1 as an example, the retrieval accuracy of our method is improved by 3.6% compared to TIRG on the Fashion200k dataset.

The remaining content of this paper is as follows. Section "Related work" is related work. Section "Method" details network architecture and related modules. Section "Experiments" is experiments. Section "Conclusion" gives conclusions.

## Related work

### Combinatorial learning

The core of combinatorial learning is a complex concept that can be extended by combining multiple simple concepts or attributes. With the complexity of retrieval background, single-modal retrieval technology has been unable to meet retrieval needs of users. In order to improve the universality of image retrieval, multi-modal image retrieval has gradually entered people's field of vision. In recent years, Visual Question Answering (VQA) has received extensive attention^12–14^.

### Combined query image retrieval methods

In the image retrieval field, many methods have been proposed to fuse image and text inputs^11,15–18^. Relationship^15^ is a relational reasoning-based method that uses CNN to extract image features, LSTM to extract text features, and then creates a set of relational features. These features are passed through an MLP, and they are averaged to obtain a combined representation. To “influence” the source image, FiLM^16^ method applies an affine transformation to the output of hidden layers in network. Another prominent method is parameter hashing^17^, where one of fully connected layers in CNN act as the dynamic parameter layer. Zhang et al.^18^ adopted Jumping Graph Attention Network to model visual and textual features. TIRG^11^ extracts reference image features by ResNet-18, extracts text features by LSTM, then reference image features and text features are combined in a 2d space using gating connections and residual connections.

### Sample distance measurement

**Figure 2:**
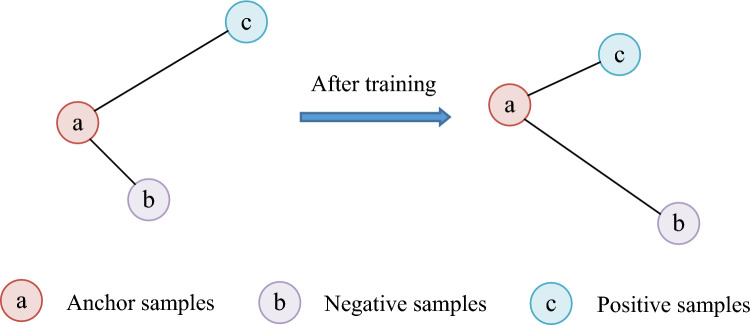
Triplet loss function for training, Euclidean distance as sample distance measurement. (Created by ‘Microsoft Office Visio 2013’ https://www.microsoft.com/zh-cn/microsoft-365/previous-versions/microsoft-vision-2013).

TIRG^11^ method uses Euclidean distance as measurement of sample distance. As shown in Fig. 2, the model trained by triplet loss function always tends to make absolute distance between anchor samples and positive samples smaller, and make absolute distance between anchor samples and negative samples larger. The above situation is likely to cause the model to blindly pursue absolute distance relationship, while ignoring angular relationship between each other, resulting in low image retrieval accuracy and poor model generalization ability. The Cosine distance is similar to the Euclidean distance. The model trained by triple loss function blindly pursues angle relationship, while ignoring absolute distance relationship between each other. Aiming at the defects of Euclidean distance and Cosine distance, we comprehensively consider both absolute distance and angle, and propose Triangle Area Triple Loss Function (TATLF). TATLF uses Triangle Area (TA) as measurement of sample distance. The method of TA measurement considers both absolute distance relationship and angle relationship. When TA performs sample distance measurement, absolute distance and angular distance both promote and restrict each other. Therefore, the trained model is more likely to produce less errors and fewer false positives than using absolute or angular distance measurement alone. Therefore, compared with the Euclidean distance or Cosine distance measurement method alone, TA method is more reasonable, and the trained model has stronger generalization ability.

### Transformer

Transformers were originally proposed for Natural Language Processing^19,20^ and Text Embedding^21^. As researchers continue to explore, Transformers can be applied not only to object detection^22^ and image classification^23–25^, but also to semantic segmentation^26^ and medical image segmentation^27,28^. Based on the powerful global modeling ability of Transformer, we introduce Transformer into combined query image retrieval. We encode the reference image through CNN and Transformer to reduce the loss of reference images information. CNN is used to extract local feature information of reference images. Meanwhile, Transformer is used to model global correlation, focusing on the edge feature information of reference images.

## Method

In this section, our research motivation and network architecture are described firstly. Then, relevant modules in the network architecture are introduced in detail. Finally, the proposed sample distance measure method TA and Triangle Area Triplet Loss Function (TATLF) are introduced.

### Research motivation

Recently, there are some defects in combined query image retrieval methods that need to be deal with: (1) Combined query image retrieval method uses Euclidean distance as the sample distance measurement method, but Euclidean distance only considers the absolute distance between samples. (2) These methods use single CNN to extract image features, which can easily lead to loss of edge feature information in the image. Based on the above considerations, the goal of this paper is to design a new sample distance measurement method, so that the model trained by triple loss function comprehensively considers the absolute distance and angle relationship between samples. Meanwhile, we combine CNN with Transformer with powerful global modeling capabilities to encode images. Therefore, our network model has the ability to capture local feature information and edge feature information of images simultaneously.

### Network architecture

**Figure 3:**
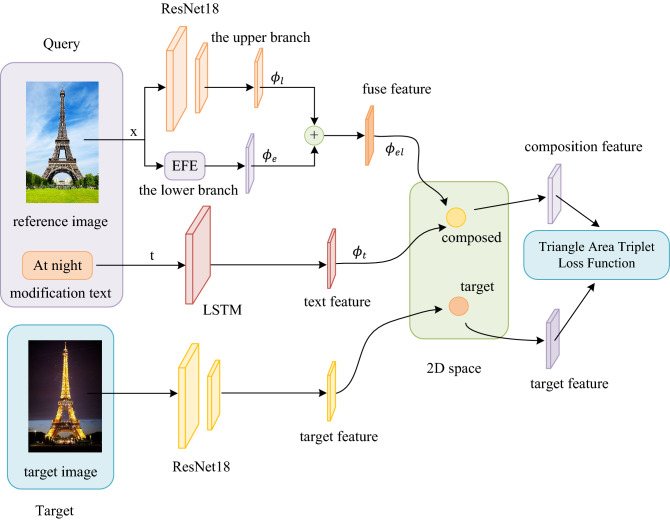
The system pipeline for training. CNN and Edge Feature Extraction (EFE) module are used to encode the reference image x. LSTM is used to extract text features. Finally, train the model via TATLF. (Created by ‘Microsoft Office Visio 2013’ https://www.microsoft.com/zh-cn/microsoft-365/previous-versions/microsoft-vision-2013).

As shown in Fig. 3, our goal is to learn the embedding space of text + image query and target images, making matching pairs (query, image) closer. On the one hand, we encode the local feature information and edge feature information of the reference image $x$ through CNN and Edge Feature Extraction (EFE) module. On the other hand, LSTM is used to extract text features.

We encode the reference image $x$ by the upper and lower branches. In the upper branch, CNN is used to encode the reference image $x$ to get 2d spatial feature vector ${f}_{img1}\left(x\right)$, which is described as Eq. (1):

1 $${f}_{img1}\left(x\right)={\phi }_{l}\in {\mathbb{R}}^{B\times C}$$

where $B$ and $C$ represent batch and the number of channels, respectively. ${\phi }_{l}$ represent local features of the reference image.

In the lower branch, the EFE module is adopted to encode the reference image $x$ to obtain 2d spatial feature vector ${f}_{img2}\left(x\right)$, which is described as Eq. (2):

2 $${f}_{img2}\left(x\right)={\phi }_{e}\in {\mathbb{R}}^{B\times C}$$

where ${\phi }_{e}$ represent edge features of the reference image.

Then, we fuse ${\phi }_{l}$ and ${\phi }_{e}$ to get ${\phi }_{el}$, it can be expressed as Eq. (3):

3 $${\phi }_{el}={\phi }_{l}+{\phi }_{e}$$

where ${\phi }_{el}$ represents the reference image fusion features.

Simultaneously, we use LSTM to extract text features ${f}_{text}\left(t\right)$, as in Eq. (4):

4 $${f}_{text}\left(t\right)={\phi }_{t}\in {\mathbb{R}}^{B\times C}$$

where ${\phi }_{t}$ is the hidden state of last time step.

Next, we combine ${\phi }_{el}$ and ${\phi }_{t}$ in a 2d space with described as Eq. (5):

5 $${\phi }_{elt}^{rg}={\omega }_{g}{f}_{gate}\left({\phi }_{el} {, \phi }_{t}\right)+{\omega }_{r}{f}_{res}\left({\phi }_{el} {, \phi }_{t}\right)$$

where ${\phi }_{elt}^{rg}$ represents the combination of text features and reference image fusion features. ${{f}_{gate} , {f}_{res}\in {\mathbb{R}}}^{B\times C\times W\times H}$ are gating features and residual features respectively, $W$ stands for width and $H$ for height. ${\omega }_{g}$ and ${\omega }_{r}$ represent their learnable weights respectively. The gating connection ${f}_{gate}\left({\phi }_{el} {, \phi }_{t}\right)$ is as follows Eq. (6):

6 $${f}_{gate}\left({\phi }_{el} {, \phi }_{t}\right)=\sigma \left({W}_{g2}*RELU\left({W}_{g1}*\left[{\phi }_{el}, {\phi }_{t}\right]\right)\right)\bigodot {\phi }_{el}$$

where $\sigma$ represents the sigmoid activation function, $\bigodot$ is element wise product, $*$ represents 2d convolution with batch normalization, ${W}_{g1}$ and ${W}_{g2}$ are 3 $\times$ 3 convolution filters. The residual connection ${f}_{res}\left({\phi }_{el} {, \phi }_{t}\right)$ is as follows Eq. (7):

7 $${f}_{res}\left({\phi }_{el} {, \phi }_{t}\right)={W}_{r2}*RELU\left({W}_{r1}*\left(\left[{\phi }_{el}{ , \phi }_{t}\right]\right)\right)$$

where ${W}_{r1}$ and ${W}_{r2}$ are 3 $\times$ 3 convolution filters.

Last but not least, we extract target image features through CNN and train our network model by means of TATLF.

### Edge feature extraction (EFE)

**Figure 4:**
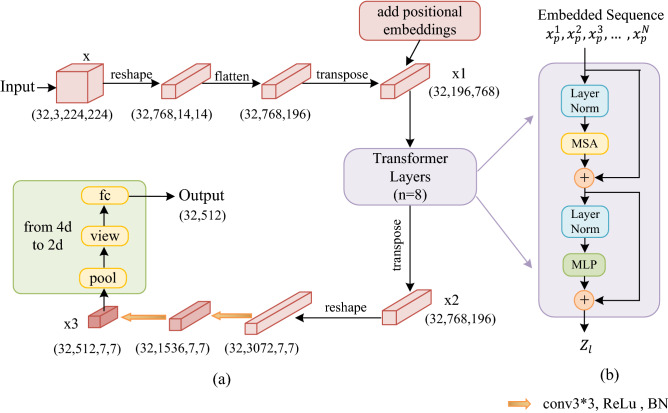
The Edge Feature Extraction (EFE) module. (**a**) The edge feature information extraction process. (**b**) The Transformer encoder module. Transformer layers is represented by n. The paper uses 4-layer or 8-layer Transformers, that is, n = 4 or n = 8. Each Transformer layer includes Layer Norm, MSA and MLP. (Created by ‘Microsoft Office Visio 2013’ https://www.microsoft.com/zh-cn/microsoft-365/previous-versions/microsoft-vision-2013).

The EFE module is shown in Fig. 4, which includes two parts (a) and (b). Specifically, as shown in Fig. 4a, we let $x\in {\mathbb{R}}^{32\times 3\times 224\times 224}$ denote the set of input tokens. Firstly, reshape $x$ to ${\mathbb{R}}^{32\times 768\times 14\times 14}$, and then compress *H* and *W* dimensions to one dimension, that is, ${\mathbb{R}}^{32\times 768\times 196}$. Finally, transpose the dimension to ${x}_{1}\in {\mathbb{R}}^{32\times 196\times 768}$. As in Eq. (8):

8 $${x}_{1}=transpose\left\{flatten\left[Re\left(x\right)\right]\right\}$$

where $Re$ represents reshape operation. $flatten$ represents compress operation, $transpose$ represents transpose operation.

Second, we input ${x}_{1}$ to Transformer layer for processing. The Transformer is set to 4 or 8 layers, and the structure of each layer is shown in Fig. 4b.

The feature vectors are first patch embedding in Transformer layer. We map the vectorized patches ${\mathrm{x}}_{p}$ into a latent D-dimensional embedding space using a trainable linear projection. To encode the patch spatial information, we add positional embeddings to the patch embeddings for preserve the positional information. As follows Eq. (9):

9 $${z}_{0}=\left[{\mathrm{x}}_{p}^{1}E; {\mathrm{x}}_{p}^{2}E; \dots ; {\mathrm{x}}_{p}^{N}E\right]+ {E}_{pos}$$

where $E\in {R}^{({P}^{2}\cdot C)\times D}$ is the patch embedding projection, and ${E}_{pos} \in {R}^{N\times D}$ denotes the position embedding.

The Transformer encoder consists of $l$ layers of Multi-head Self Attention (*MSA*) and Multi-Layer Perceptron (*MLP*) blocks. Output of the *l*-th layer can be written as follows Eqs. (10) and (11):

10 $${z}_{l}^{^{\prime}}=MSA\left(LN\left({z}_{l-1}\right)\right)+{z}_{l-1}$$

11 $${z}_{l}=MLP\left(LN\left({z}_{l}^{^{\prime}}\right)\right)+{z}_{l}^{^{\prime}}$$

where $LN\left(\cdot \right)$ denotes the layer normalization operator and ${z}_{l}$ is the encoded image representation. When this paper set Transformer layers to 8, the final output after Transformer layers is ${z}_{8}\in {\mathbb{R}}^{32\times 196\times 768}$. The output of Transformer is transposed to get ${x}_{2}\in {\mathbb{R}}^{32\times 768\times 196}$.

Next, we reshape ${x}_{2}$ to ${\mathbb{R}}^{32\times 3072\times 7\times 7}$, and pass through two convolutional layers to convert the number of channels to 512. The purpose is keeping the number of output channels of image features consistent with the number of output channels of text features, so as to facilitate feature combination. The output after two convolution operations is ${x}_{3}\in {\mathbb{R}}^{32\times 512\times 7\times 7}$. As in Eq. (12):

12 $${x}_{3}={W}_{2}\left\{{W}_{1}\left[Re\left({x}_{2}\right)\right]\right\}$$

where ${W}_{1}$, ${W}_{2}$ represent 3 $\times$ 3 convolutions. ${W}_{1}$∈${R}^{3072\times 1536}$, 3072 is the channels of input and 1536 is the channels of output. ${W}_{2}$∈${R}^{1536\times 512}$, the 1536 is the channels of input and 512 is the channels of output.

Finally, we perform dimensional compression to compress 4d image features to 2d for effortless combination with text features. The compression process adopts $pool$, $view$ and full connection operation. As in Eq. (13):

13 $${x}_{out}=FC\left\{view\left[pool\left({x}_{3}\right)\right]\right\}$$

where $FC$ represents full connection operation.

In the EFE module, Transformer is used in our network, which makes the network pay attention to the edge feature information that CNN is easy to ignore. Therefore, CNN and EFE module are used for feature extraction of reference images in our network, which has the ability to jointly model edge information and local information. Thus, our network has outstanding performance than single CNN.

### The proposed sample distance measurement method: TA

**Figure 5:**
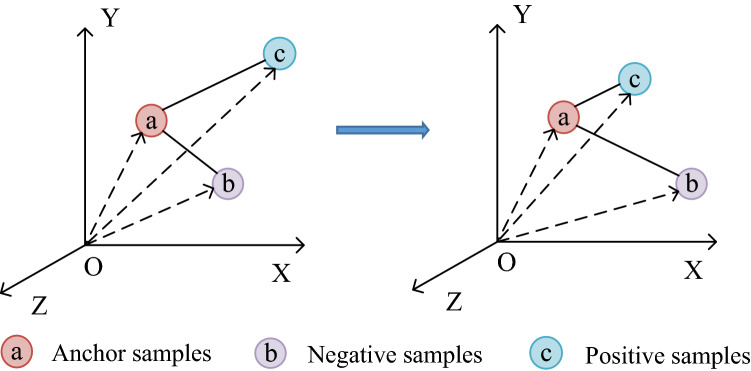
Triangle Area Triplet Loss Function for training, TA is adopted as sample distance measurement. (Created by ‘Microsoft Office Visio 2013’ https://www.microsoft.com/zh-cn/microsoft-365/previous-versions/microsoft-vision-2013).

TA is a completely new sample measurement proposed in this paper. The main idea of TA is area measurement, that is, Triangle Area is adopted to measure sample distance. As shown in Fig. 5, the sample distance between anchor sample (a) and negative sample (b) is represented by the area of triangle (Oab). Similarly, the sample distance between anchor sample (a) and positive sample (c) is represented by the area of triangle (Oac). Triangle Area not only considers the absolute distance between samples, but also included angle. More importantly, Triangle Area perfectly utilizes optimal weights of the two, and there is unnecessary to consider optimal weights of the two separately. As shown in Fig. 5, model is trained with triple loss function, and TA is adopted as sample distance measurement. In the training process, TA is only necessary to make the area of triangle (Oac) enclosed by anchor sample (a) and positive sample (c) smaller, and make the area of triangle (Oab) enclosed by anchor sample (a) and negative sample (b) larger. In this case, the trained model comprehensively considers distance relationship and included angle relationship. In this way, TA makes sample test more rational, effectively enhances generalization ability of model and greatly improves accuracy of image retrieval. The calculation method of TA is shown in Eqs. (17) and (18).

### Triangle area triplet loss function (TATLF)

We suppose have a training minibatch of *B* queries, ${\psi }_{i}={f}_{combine}\left({x}_{i}^{query}, {t}_{i}\right)$ is the final modified representation of image text query, and ${\phi }_{i}^{+}={f}_{img}\left({x}_{i}^{target}\right)$ is the representation of target image of that query. We create a set ${\mathcal{N}}_{i}$ consisting of one positive example ${\phi }_{i}^{+}$ and $K-1$ negative examples ${\phi }_{1}^{-}$,… ,${\phi }_{k-1}^{-}$ (by sampling from the minibatch ${\phi }_{j}^{+}$ where $j$ is not $i$ ). We repeat this *M* times, denoted as ${\mathcal{N}}_{i}^{m}$, to evaluate every possible set. (The maximum value of *M* is $\left(\genfrac{}{}{0pt}{}{B}{K}\right)$, but we often use a smaller value for tractability.)

We use the following Triangle Area Triplet Loss Function (TATLF), as shown in Eq. (14):

14 $$L=\frac{-1}{MB}\sum_{i=1}^{B}\sum_{m=1}^{M}\mathrm{log}\left\{\frac{\mathrm{exp}\left\{TA\left({\psi }_{i} , {\phi }_{i}^{+}\right)\right\}}{{\sum }_{{\phi }_{j}\in {\mathcal{N}}_{\mathrm{i}}^{m}}\mathrm{exp}\left\{TA\left({\psi }_{i} , {\phi }_{j}\right)\right\}}\right\}$$

When dataset is small (MIT-States), we set $K=2$, $M=B-1$, the following loss function Eq. (15) can be obtained from Eq. (14):

15 $$L=\frac{1}{MB}\sum_{i=1}^{B}\sum_{m=1}^{M}\mathrm{log}\left\{1+\mathrm{exp}\left\{TA\left({\psi }_{i} , {\phi }_{i,m}^{-}\right)-TA\left({\psi }_{i} , {\phi }_{i}^{+}\right)\right\}\right\}$$

When dataset is large (Fashion200k), we set $K=B$, $M=1$, the following loss function Eq. (16) can be obtained from Eq. (14):

16 $$L=\frac{1}{B}\sum_{i=1}^{B}-\mathrm{log}\left\{\frac{\mathrm{exp}\left\{TA\left({\psi }_{i} , {\phi }_{i}^{+}\right)\right\}}{{\sum }_{j=1}^{B}\mathrm{exp}\left\{TA\left({\psi }_{i} , {\phi }_{j}^{+}\right)\right\}}\right\}$$

where $TA$ is a new sample distance measurement method proposed in this paper, it is described in the previous section. Depending on the size of dataset, $TA$ has two forms of computation.

When dataset is small (MIT-States), the calculation method of $TA$ is shown in Eq. (17):

17 $${TA\left({\psi }_{i} , {\phi }_{i}\right)}_{s}=\frac{1}{4}{\left(\left|{\psi }_{i}\right|\left|{\phi }_{i}\right|\right)}^{2}\left\{1-{\left(\frac{{\psi }_{i}\cdot {\phi }_{i}}{\left|{\psi }_{i}\right|\left|{\phi }_{i}\right|}\right)}^{2}\right\}$$

When dataset is large (Fashion200k), the calculation method of $TA$ is shown in Eq. (18):

18 $${TA\left({\psi }_{i} , {\phi }_{i}\right)}_{l}=\frac{1}{2}\left|{\psi }_{i}\right|\left|{\phi }_{i}\right|\sqrt{1-{\left(\frac{{\psi }_{i}\cdot {\phi }_{i}}{\left|{\psi }_{i}\right|\left|{\phi }_{i}\right|}\right)}^{2}}$$

where ${\psi }_{i}$ generally refers to feature vector after query images and texts are combined, and ${\phi }_{i}$ generally refers to feature vector of positive or negative samples.

In conclusion, when dataset is small (MIT-States), our experiment uses Eqs. (15) and (17) to train model. The square of Triangle Area is used to measure sample distance, which will make difference between samples larger, convergence is slower, preventing model from overfitting. When dataset is large (Fashion200k), our experiment uses Eqs. (16) and (18) to train model. The Triangle Area is used as measurement of sample distance, so that difference between samples will not increase, convergence is faster and training time cost is effectively saved.

## Experiments

### Experimental setups

The experiment uses two available public datasets Fashion-200 k and MIT-States. The pytorch framework is used in our experiments, the version of python is 3.6. We use ResNet18 and Transformer (output feature size = 512) as our image encoder and LSTM with random initial weights (hidden size = 512) as our text encoder. The evaluation metric of retrieval is recall (R@K), which is calculated as percentage of test queries. The values of k for large (Fashion-200 k) and small (MIT-States) datasets are set to 1, 10, 50 and 1, 5, 10, respectively. And for the training of the model is using SGD optimizer with learning rate of 0.01, momentum of 0.9 and weight decay of 1e-6. For both Fashion-200 k and MIT-States datasets, we have default batch size of 32 and the training runs for 160 k iterations. All experiments were performed using a single NVIDIA Corporation GV100 [TITANV] GPU.

### Datasets

MIT-States^29^ dataset contains about 60 k images, each image is described by a noun and an adjective, the noun represents the category, and the adjective represents the state. There are 245 nouns in the dataset, of which 196 are used for training and 49 are used for testing. This split ensures that the algorithm can learn unseen combinations of nouns. An input image (say “city”) is sampled and a text query asks to change the state to “ancient”. The algorithm is considered successful if it retrieves the correct target image (“ancient city”) from the pool of all test images.

Fashion200k^30^ contains about 200 k images. There are five fashion categories in the dataset, namely: pants, skirts, dresses, tops and jackets. Each image has a human annotated title, such as “pink mandarin collar jacket.” The training set contains 172,049 images and the test set contains 29,789 images.

### Evaluation metrics

Consistent with the baseline^11^, we set recall (R@K) as the evaluation metrics in this paper. Recall is defined as the percentage of images predicted to be positive samples to the total number of images in all positive samples. Specifically, the calculation method of recall (R@K) is shown in Eqs. (19) and (20):

19 $$recall=\frac{TP}{TP+FN}$$

20 $$R@K=\frac{1}{n}\sum_{i=1}^{k}(score)$$

where $TP$ is true-positive, $FN$ is false-negative. $K$ is the total number of images returned, $n$ is the total number of pictures of all positive samples ($TP+FN$), the value of $score$ is either 1 (when prediction is positive sample) or 0 (when prediction is negative sample), $\sum_{i=1}^{k}(score)$ is the number of images predicted to be positive samples ($TP$), $\sum_{i=1}^{k}(score)\in \left[0,k\right]$.

### Experimental results

This paper compares some classical algorithms, including Show and Tell^31^, Parameter Hashing^17^, Attribute as Operator^32^, Relationship^15^, FiLM^16^, TIRG^11^, Zhang et al.^18^.

The retrieval performance comparison results on the Fashion200k dataset are shown in Table 1. The best number is in bold, the next best number is underlined. From Table 1, it can be clearly seen that our algorithm outperforms other algorithms. Specifically, compared with Zhang et al.^18^, our method improves by 0.4% on R@1, 1.6% on R@10, and 0.5% on R@50, respectively. Compared with baseline TIRG^11^, our method improves by 3.6% on R@1, 4.3% on R@10, and 2.4% on R@50, respectively. The above experimental results demonstrate excellent performance of our proposed method.

**Table 1:** Comparison results of retrieval performance on Fashion200k dataset.

Method	R@1	R@10	R@50
Han et al.^30^	6.3	19.9	38.3
Image only^11^	3.5	22.7	43.7
Text only^11^	1.0	12.3	21.8
Concatenation^11^	11.9 ± 1.0	39.7 ± 1.0	62.6 ± 0.7
Show and Tell^31^	12.3 ± 1.1	40.2 ± 1.7	61.8 ± 0.9
Param Hashing^17^	12.2 ± 1.1	40.0 ± 1.1	61.7 ± 0.8
Relationship ^15^	13.0 ± 0.6	40.5 ± 0.7	62.4 ± 0.6
FiLM^16^	12.9 ± 0.7	39.5 ± 2.1	61.9 ± 1.9
TIRG^11^	14.1 ± 0.6	42.5 ± 0.7	63.8 ± 0.8
Zhang et al.^18^	17.3 ± 0.6	45.2 ± 0.9	65.7 ± 0.8
**Ours**	**17.7 ± 0.6**	**46.8 ± 0.6**	**66.2 ± 0.9**

The retrieval performance comparison results on the MIT-States dataset are shown in Table 2. The best number is in bold, the next best number is underlined. From Table 2, it can be clearly seen that our algorithm outperforms other algorithms. Specifically, compared with Zhang et al.^18^, our method improves by 0.1% on R@5. Compared with baseline TIRG^11^, our method improves by 1.0% on R@1, 1.4% on R@5, and 1.2% on R@10, respectively. The above experimental results demonstrate excellent performance of our proposed method.

**Table 2:** Comparison results of retrieval performance on MIT-States dataset.

Method	R@1	R@5	R@10
Image only^11^	3.3 ± 0.1	12.8 ± 0.2	20.9 ± 0.1
Text only^11^	7.4 ± 0.4	21.5 ± 0.9	32.7 ± 0.8
Concatenation^11^	11.8 ± 0.2	30.8 ± 0.2	42.1 ± 0.3
Show and Tell^31^	11.9 ± 0.1	31.0 ± 0.5	42.0 ± 0.8
Att. as Operator^32^	8.8 ± 0.1	27.3 ± 0.3	39.1 ± 0.3
Relationship^15^	12.3 ± 0.5	31.9 ± 0.7	42.9 ± 0.9
FiLM^16^	10.1 ± 0.3	27.7 ± 0.7	38.3 ± 0.7
TIRG^11^	12.2 ± 0.4	31.9 ± 0.3	43.1 ± 0.3
Zhang et al.^18^	**14.2 ± 0.5**	33.2 ± 0.5	**45.3 ± 0.6**
**Ours**	13.2 ± 0.8	**33.3 ± 1.0**	44.3 ± 0.9

**Figure 6:**
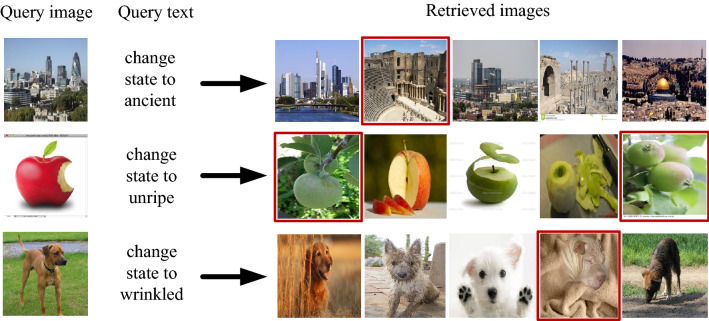
Qualitative Results (R@5): Retrieval examples from MIT-States Dataset. (Created by ‘Microsoft Office Visio 2013’ https://www.microsoft.com/zh-cn/microsoft-365/previous-versions/microsoft-vision-2013).

Qualitative Results: The Qualitative Results on the MIT-States dataset is shown in Fig. 6. Query image in the first row is “city”, meanwhile, state of the query image is modified to “ancient” through text, images on the right are retrieved images. Target images are marked with red border. Query image in the second row is “apple”, meanwhile, state of the query image is modified to “unripe” through text, images on the right are retrieved images. Target images are marked with red border. Query image in the third row is “dog”, meanwhile, state of the query image is modified to “wrinkled” through text, images on the right are retrieved images. Target images are marked with red border.

### Ablation studies

#### On the influence of EFE module (mainly refers to transformer) and TATLF

As shown in Tables 3 and 4, “TIRG + EFE” refers to adding EFE module on the basis of TIGR. “TIRG + TATLF” refers to training TIRG by Triangle Area Triplet Loss Function. “**Ours**” refers to training the network model proposed in this paper by Triangle Area Triplet Loss Function. Compared with baseline TIRG^11^ on the Fashion200k dataset, take R@1 as an example, retrieval accuracy of “TIRG + EFE” is improved by 2.0%, retrieval accuracy of “TIRG + TATLF” is improved by 1.0%, retrieval accuracy of “**Ours**” is improved by 3.6%. Compared with baseline TIRG^11^ on the MIT-States dataset, take R@1 as an example, retrieval accuracy of “TIRG + EFE” is improved by 0.4%, retrieval accuracy of “TIRG + TATLF” is improved by 0.9%, retrieval accuracy of “**Ours**” is improved by 1.0%.

**Table 3:** Ablation studies of our method on Fashion200k dataset.

Method	R@1	R@10	R@50
TIRG^11^	14.1 ± 0.6	42.5 ± 0.7	63.8 ± 0.8
TIRG + EFE(n = 8)	16.1 ± 0.7	44.5 ± 0.9	65.6 ± 0.8
TIRG + TATLF	15.1 ± 0.5	45.9 ± 0.7	66.1 ± 0.9
**Ours**	17.7 ± 0.6	46.8 ± 0.6	66.2 ± 0.9

**Table 4:** Ablation studies of our method on MIT-States dataset.

Method	R@1	R@5	R@10
TIRG^11^	12.2 ± 0.4	31.9 ± 0.3	43.1 ± 0.3
TIRG + EFE(n = 4)	12.6 ± 0.5	32.5 ± 0.4	43.9 ± 0.5
TIRG + TATLF	13.1 ± 0.3	32.2 ± 0.6	43.5 ± 0.5
**Ours**	13.2 ± 0.8	33.3 ± 1.0	44.3 ± 0.9

Visible from above, using the ability of Transformer to model global correlation can focus on edge feature information that CNN is easy to ignore, which can reduce the loss of edge feature information of the reference image. Therefore, the retrieval accuracy is improved.

Compared with the previous triple loss function, TATLF uses Triangle Area as measurement between samples. Triangle Area not only considers the absolute distance between samples, but also considers the angle between samples, which makes the trained model have stronger generalization ability.

#### On the influence of transformer layers

As shown in Table 5, on the Fashion200k dataset, the retrieval performance of “Ours(n = 8)” is better than that of “Ours(n = 4)”. Specifically, take R@1 as an example, the retrieval accuracy of “Ours(n = 8)” is improved by 2.1% compared to “Ours(n = 4)”. As shown in Table 6, on the MIT-States dataset, the retrieval performance of “Ours(n = 4)” is better than that of “Ours(n = 8)”. Specifically, take R@1 as an example, the retrieval accuracy of "Ours(n = 4)" is improved by 0.4% compared to “Ours(n = 8)”. It is obvious that increasing the number of Transformer layers can improve the retrieval performance on large dataset (Fashion200k). But for small dataset (MIT-States), Increasing the number of Transformer layers will cause information redundancy, which will result in an insignificant increase in retrieval performance.

**Table 5:** Ablation studies of Transformer layers on Fashion200k dataset.

Method	R@1	R@10	R@50
Ours(n = 4)	15.6 ± 0.7	44.7 ± 0.9	65.2 ± 0.4
Ours(n = 8)	17.7 ± 0.6	46.8 ± 0.6	66.2 ± 0.9

**Table 6:** Ablation studies of Transformer layers on MIT-States dataset.

Method	R@1	R@5	R@10
Ours(n = 4)	13.2 ± 0.8	33.3 ± 1.0	44.3 ± 0.9
Ours(n = 8)	12.8 ± 0.4	32.5 ± 0.5	43.6 ± 0.8

#### On the influence of loss function

As shown in Tables 7 and 8, “Ours(Ed)” refers to training our network model by Triplet Loss Function, Euclidean distance as sample distance measurement. “Ours(Cd)” refers to training our network model by Triplet Loss Function, Cosine distance as sample distance measurement. “**Ours**” refers to training our network model by Triangle Area Triplet Loss Function. “**Ours**” has better retrieval performance than “Ours(Ed)” and “Ours(Cd)”. Specifically, take R@1 as an example, on the Fashion200k dataset, the retrieval accuracy of “**Ours**” improved by 1.6% compared to “Ours(Ed)” and 2.0% compared to “Ours(Cd)”. On the MIT-States dataset, the retrieval accuracy of “**Ours**” increased by 0.6% compared to “Ours(Ed)”, and increased by 0.3% compared to “Ours(Cd)”. The reason is that the model trained by TATLF not only considers the absolute distance between samples, but also considers the angle between samples, which makes the sample testing more reasonable. Thus, retrieval performance of the model is improved.

**Table 7:** Ablation studies of loss function on Fashion200k dataset.

Method	R@1	R@10	R@50
Ours(Ed)	16.1 ± 0.7	44.5 ± 0.9	65.6 ± 0.8
Ours(Cd)	15.7 ± 0.5	44.7 ± 0.6	65.9 ± 1.1
**Ours**	17.7 ± 0.6	46.8 ± 0.6	66.2 ± 0.9

**Table 8:** Ablation studies of loss function on MIT-States dataset.

Method	R@1	R@5	R@10
Ours(Ed)	12.6 ± 0.5	32.5 ± 0.4	43.9 ± 0.5
Ours(Cd)	12.9 ± 0.7	32.4 ± 0.5	43.7 ± 0.8
**Ours**	13.2 ± 0.8	33.3 ± 1.0	44.3 ± 0.9

### Discussion

Through the above comparative experiments and ablation studies, we find that combined query image retrieval has great challenges in case of capture reference image feature information and sample distance measurement. But the network we designed achieved great results. Compared with other comparison networks, our network has stronger ability to capture image feature information, and the sample distance metric is more reasonable. However, our method still has room for optimization. In future research, we will try to reduce the amount of network parameters without affecting retrieval performance.

## Conclusion

In this paper, we propose Triangle Area Triple Loss Function (TATLF), which adopts Triangle Area (TA) as measurement of sample distance. The advantage of the model trained by TATLF not only considers the distance relationship between samples, but also considers the angle relationship. As a result, retrieval performance of the model is improved. Furthermore, we combine CNN with Transformer. It allows our network model to have the ability to jointly model local information and edge information, which has better performance than single CNN. Extensive experiments on two public datasets, Fashion200k and MIT-States, confirm excellent performance of our proposed method.

## Data Availability

The MIT-States and Fashion200k datasets are openly available at: http://web.mit.edu/phillipi/Public/states_and_transformations/index.html (accessed on 27 March 2022) and https://github.com/xthan/fashion-200k (accessed on 27 March 2022).
